# Distribution of enteropathogenic *Yersinia* spp. and *Salmonella* spp. in the Swedish wild boar population, and assessment of risk factors that may affect their prevalence

**DOI:** 10.1186/s13028-018-0395-3

**Published:** 2018-07-03

**Authors:** Axel Sannö, Thomas Rosendal, Anna Aspán, Annette Backhans, Magdalena Jacobson

**Affiliations:** 1 Department of Clinical Sciences, Swedish University of Agricultural Sciences (SLU), Box 7054, 750 07 Uppsala, Sweden; 2 Department of Disease Control and Epidemiology, National Veterinary Institute, Uppsala, Sweden; 3 Department of Microbiology, National Veterinary Institute, Uppsala, Sweden

**Keywords:** Human enteropathogens, Risk factors, Sweden, *Salmonella* spp., Wild boars, *Yersinia enterocolitica*, *Yersinia pseudotuberculosis*, Zoonotic diseases

## Abstract

**Background:**

Pure Eurasian wild boars and/or hybrids with domestic pigs are present in the wild on most continents. These wild pigs have been demonstrated to carry a large number of zoonotic and epizootic pathogens such as *Salmonella* spp., *Yersinia enterocolitica* and *Y*. *pseudotuberculosis*. Wild boar populations throughout Europe are growing and more and more wild boar meat is being consumed, the majority within the homes of hunters without having passed a veterinary inspection. The aim of this study was to investigate if factors such as population density, level of artificial feeding, time since establishment of a given population, and the handling of animal by-products from slaughtered animals could influence the presence of these pathogens in the wild boar.

**Results:**

In total, 90 wild boars from 30 different populations in Sweden were sampled and analysed using a protocol combining pre-cultivation and PCR-detection. The results showed that 27% of the sampled wild boars were positive for *Salmonella* spp., 31% were positive for *Y. enterocolitica* and 22% were positive for *Y. pseudotuberculosis*. In 80% of the sampled populations, at least one wild boar was positive for one of these enteropathogens and in total, 60% of the animals carried at least one of the investigated enteropathogens. The presumptive risk factors were analysed using a case–control approach, however, no significant associations were found.

**Conclusion:**

Human enteropathogens are commonly carried by wild boars, mainly in the tonsils, and can thus constitute a risk for contamination of the carcass and meat during slaughter. Based on the present results, the effect of reducing population densities and number of artificial feeding places might be limited.

## Background

Pure Eurasian wild boars and/or hybrids with domestic pigs are present in the wild on most continents. These wild pigs may carry a large number of zoonotic and epizootic pathogens [1] and recent studies have focused on the presence of the commonly occurring zoonotic agents *Trichinella* spp., *Salmonella* spp., *Yersinia pseudotuberculosis, Y. enterocolitica,* hepatitis E virus and *Toxoplasma gondii* [2–5]. Human enteropathogenic *Y. enterocolitica* and *Y. pseudotuberculosis* have been isolated from domestic pigs [6, 7] as well as in wild boars and rodents [3, 8, 9]. Thus, wild boars, rodents and birds [10] may act as vectors and constitute a risk for farms with domestic pigs through, e.g. contaminated feed [11]. The infections have also been found in several other animal species [12] sharing habitat with wild boars.

However, few studies have addressed the risk factors associated with these infections in wild boars, although proximity to *Salmonella*-infected grazing cattle has been identified as a risk for sympatric wild boars to become infected by cattle-associated *Salmonella* spp. [13]. Furthermore, the crowding that may occur at artificial feeding places especially during the winter has been suggested to increase the risk for transmission of pathogens such as *Salmonella* spp. [14–16]. Factors that hypothetically may influence the presence of pathogens in wild boars are population densities, time since establishment of the local population [17], and the use of artificial feeding places.

The recently established Swedish wild boar population is unevenly distributed in the southern part of Sweden covering 13 counties, and with large variations in densities and hunting management [18]. Similarly to other European countries, the wild boar population has increased during the past decade and spread to new areas. Thus the annual hunting bag in Sweden has increased 10-fold [19]. According to the European legislation (EC No 853/2004) [20], no wild boars or parts thereof are allowed on the market without passing a veterinary inspection at a wild game handling establishment, and the animal by-products are destroyed in accordance with EC No 1069/2009 [21]. However, only 15% of harvested wild boars pass through such an establishment [22]. Most of the wild boar meat is thus handled and consumed within the homes of the hunters, in which case, a veterinary inspection is not mandatory and all by-products from hunted wild game may be left in the forest (EC No 853/2004). Thus, management of waste from shot and slaughtered animals might also be a factor influencing the presence of zoonotic agents.

The aim of this study was to investigate if factors such as population density, level of artificial feeding, time since establishment of a given population, and the handling of animal by-products from slaughtered animals could influence the presence of *Salmonella* spp., *Y. pseudotuberculosis* and *Y. enterocolitica* in the wild boar.

## Methods

### Experimental design

**Fig. 1:**
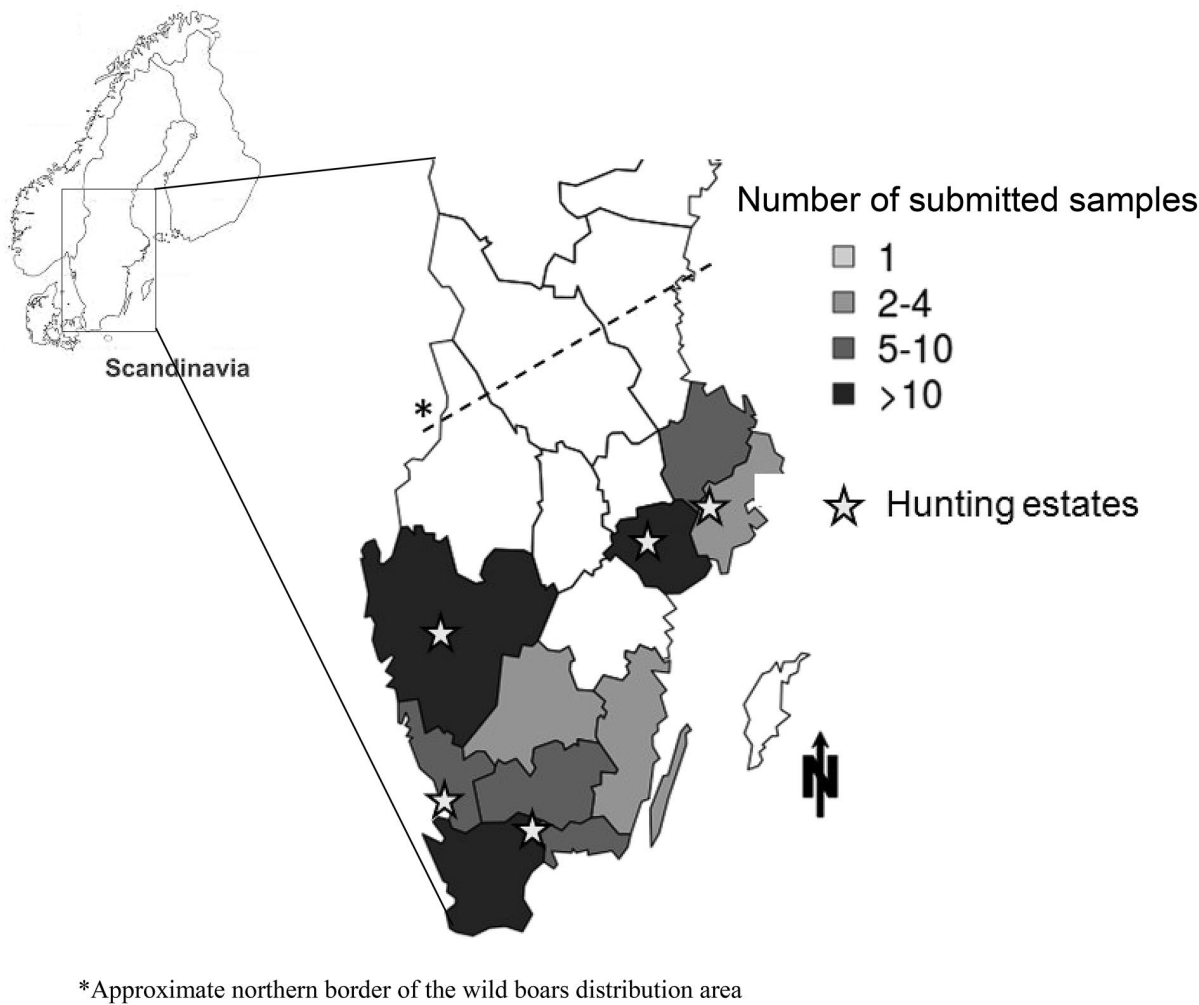
Distribution of the animals sampled and investigated for the presence of human enteropathogens. Animals from ten out of the 13 counties of the southern part of Sweden, where wild boars are present, were obtained. Hunters in all 13 counties were requested to participate

An established network of hunters organized by the Swedish hunters’ association in 13 counties in southern Sweden with wild boars present in various population densities, were asked to submit samples from shot wild boars (Fig. 1). In addition, sampling was performed on five commercial hunting estates with access to wild game handling establishments. The estates were chosen based on their geographical location and the owners’ willingness to participate in the study.

The aim was to obtain samples from 50 animals from population areas with an extensively managed, low density, newly established population not using artificial feeding places (population category 1); samples from 50 animals from populations with an intensely managed, high density, well established population using artificial feeding places (population category 2), and samples from 50 animals from commercially managed hunting estates with high population density, high level of artificial feeding and well established population (population category 3). Further, information on the handling of slaughter waste from wild boars shot by private hunters were requested. By legislation, the hunting estates do not leave any slaughter waste in the forest. Sample size calculations were completed according to standard methods for comparing proportions corrected for clustering [23]. This indicated that the planned sampling would be able to detect a difference of 18% in the variable of interest. In total, 220 sampling kits including instructions for sampling, along with a short questionnaire on the characteristics of the population and the animals sampled, were distributed. Since reliable methods for estimations of the wild boar population densities are not available, annual hunting bags were used as a proxy for population density.

### Sampling

The hunters were instructed to collect samples from shot wild boars including tonsils, one mesenteric lymph node, and faeces. The samples were frozen in 15 mL Falcon tubes (Sarstedt AG & Co, Nümbrecht, Germany) and sent on ice by ordinary mail to the laboratory. All samples were kept frozen at − 20 °C until analysis (maximum 18 months storage). The questionnaire was to be filled out and sent in with the samples and included questions on sex, weight and time of sampling of each wild boar, and information on the population characteristics for the population in the area where the wild boars were shot.

The questions on population characteristics [24] are given in Table 1.

**Table 1:** The distribution of the 30 wild boar populations in the respective risk factor category, based on the answers in a questionnaire that accompanied the samples

Risk factors	Number (%) of populations in each category
Feeding intensity (feeding places/10 km^2^)	
< 3	7 (23.3)
3–5	15 (50.0)
5–10	3 (10.0)
> 10	5 (16.7)
Years since establishment of population (years)	
< 3	1 (3.3)
3–5	3 (10.0)
5–7	4 (13.3)
7–10	5 (16.7)
> 10	17 (56.7)
Yearly harvest/10 km^2^ (animals)	
< 5	6 (20.0)
5–15	12 (40.0)
15–30	3 (10.0)
30–50	5 (16.7)
> 50	4 (13.3)
Handling of slaughter waste	
Made unavailable for wild boars	11 (36.7)
Left out in the forest	19 (63.3)

### Sample preparation

The samples were thawed, inspected macroscopically, trimmed from fat, muscle and connective tissue, and cut into 1–3 mm^3^ pieces. A maximum of 1.5 g of tissue or faeces was put in Falcon tubes with buffered peptone water (BPW) to a final dilution of 1:10 (w:w). The tubes were vortexed briefly and incubated for 20 ± 2 h. A bacterial inoculation loop (approximately 10 µL) from the top layer of the broth was spread onto Cefsulodin–Irgasan–Novobiocin (CIN), Brilliant Green (BG) and xylose–lysine–desoxycholate (XLD) agar plates (Oxoid, Hampshire, UK) before being incubated for 20 ± 2 h (30 °C on CIN-agar and 37 °C on BG- and XLD-agar). Small, white to greyish colonies with a red “bulls-eye” on CIN- [25], black colonies on XLD-, and red colonies on BG-agar [26] were collected (10 µL). If colonies with typical appearance were absent, 1–2 loops of a variety of colonies was collected at random from each plate. The material from the BG and XLD-agar plates were pooled in one tube containing 4 mL of Brain Heart Infusion broth (BHI; Oxoid, Hampshire, UK) and the material from the CIN-agar plates was dissolved in another tube to facilitate subsequent cultivation and confirmation of the results (data not shown). To prepare a template for polymerase chain reaction (PCR), the tubes were vortexed and 100 µL from each of the two tubes was pooled in a 1.5 mL Eppendorf tube and centrifuged at 12,000×*g* for 5 min to create a pellet of colony material. The supernatant was discarded and 200 µL of Instagene Matrix^®^ (BioRad, Hercules, CA, USA) was added. The mixture was incubated according to the manufacturer’s instructions during agitation at 500 rpm for 15 min at 56 °C followed by 5 min at 95 °C, before being centrifuged at 12,000×*g* for 3 min. The supernatant was used as template in the PCR.

The remaining BHI suspension was frozen (− 80 °C) in duplicate in 2 mL tubes with 15% glycerol.

### PCR

All samples were analysed by PCR performed in a 7500 Fast Real-Time PCR System (Applied Biosystems, Foster City, CA, USA) and analysed in duplicate. Based on our previous experience [3], a C_t_ (cycle threshold) value below 40 was considered as a positive result. If only one of the duplicates was deemed positive, the analysis was repeated once. The results was finally deemed as positive if three out of four results were deemed as positive following re-analysis.

The PCR for *Y. enterocolitica* and *Y. pseudotuberculosis* targeted the chromosomally encoded attachment and invasion (*ail*) gene. A real-time PCR protocol modified from Lambertz et al. [27, 28] with primers and a *Taq*Man-MGB probe manufactured at Eurofins MWG Operon, Germany, was applied. The PCR mixture consisted of 7.5 µL Perfecta Q-PCR toughmix Low-ROX (Quanta Biosciences, Gaithersburg, Maryland, USA), 750 nM of each primer, 150 nM of the probe, 2 µL template and was adjusted with ddH_2_O (Sigma Aldrich) to a total volume of 15 µL. The PCR cycling conditions consisted of an initial denaturation of the template DNA at 95 °C for 6 min, followed by 45 cycles at 95 °C for 15 s and at 60 °C for 60 s. The reference strains CCUG 45643 (4/O:3) for *Y. enterocolitica* and the reference strain CCUG 5855 for *Y. pseudotuberculosis* was used as positive controls and ddH_2_O was used as a negative control.

In the analyses for the presence of *Salmonella* spp., primers and a *Taq*Man probe targeting the invasion (*invA*) gene were used (Thermo Scientific Biopolymers, Ulm, Germany; [29]. Probes were labelled with 6-carboxyfluorescein (FAM) and Black Hole Quencher-1 (BHQ-1). A modified protocol based on the work by Hoorfar et al. [29] was used, with a PCR mixture that consisted of 7.5 µL Perfecta Q-PCR toughmix Low-ROX (Quanta Biosciences), 500 nM of each primer, 100 nM of the probe, 2 µL of the template and adjusted with ddH_2_O (Sigma Aldrich) to a total volume of 15 µL. The PCR cycling conditions consisted of an initial denaturation at 95 °C for 6 min, followed by 45 cycles at 95 °C for 15 s and at 60 °C for 60 s. The reference strain *Salmonella* Typhimurium CCUG 31969 was used as a positive control and ddH_2_O as a negative control.

All negative samples were rerun with an internal positive control (IPC) where EXO IPC/VIC Mix including 0.3 µL 1 × EXO IPC DNA (Life technologies, Grand Island, New York, USA) were added to the original PCR mixture, to investigate the possible presence of PCR-inhibitors.

### Statistical analysis

The correlation between feeding intensity and population was tested using Spearman’s rank correlation test at the population level.

Associations were tested using logistic regression. Individuals that were PCR-positive for any of the microorganisms were used as cases and PCR-negative individuals were used as controls. The potential risk factors were assessed by classifying the answers obtained in the survey as follows:

- Population density.
  - High density > 30 wild boars were shot annually per 10 km^2^.
  - Low density < 30 wild boars were shot annually per 10 km^2^ (Based on findings by Engelmann et al. [30]).
- Feeding intensity.
  - High intensity > 5 feeding places were used per 10 km^2^.
  - Low intensity < 5 feeding places were used per 10 km^2^ (Based on findings by Karlsson [31]).
- Population age.
  - Well established: wild boars had been present in the area for 10 years or longer.
  - Less established: wild boars had been present for < 10 years (Based on findings by Engelmann et al. [30]).
- Handling of slaughter waste.
  - Poor handling: slaughter waste was left out in the forest.
  - Good handling: slaughter waste was destroyed or in other ways made unavailable to wild boars and other wildlife.

The referent groups were set as “low population density”, “low intensity feeding”, “less established population” and “good handling of slaughter waste”.

The association between each of the above risk factors and the status of the animals for each pathogen was tested using a logistic regression model with a random effect to adjust for repeated sampling within each local population. The analysis was performed using the lme4 package [32] version 4.1.1-12 in R version 3.3.2 [33]. Confidence intervals of 95% were calculated around the estimates of the odds ratios (OR) for each risk factor from the standard errors of the models. Odds ratio for the different risk factors were calculated for the presence of each pathogen separately and for the presence of any of the pathogens in an individual animal.

## Results

### Sampling

During the years 2014–2016, a total of 354 samples from 90 wild boars, representing ten out of the thirteen counties (Fig. 1), were obtained (four samples were obtained per individual, from four individuals six tonsil samples were missing). Mesenteric lymph nodes and faeces were submitted from all individuals. However, from 21 individuals one or both of the samples marked as “Tonsil” were deemed to be other tissue from the throat region, such as submandibular lymph nodes, parotic glands, muscle, or parts of mucous membranes and tongue (Table 2). Samples from 47 animals were submitted from private hunters while 43 animals were sampled on five different hunting estates by the first author. All samples were accompanied by a questionnaire filled out accordingly. In total, the samples originated from 30 different populations (defined as coming from within an area of approximately < 10 km^2^, the approximate home-range for groups of wild boar [24]). All animals were divided into 3 different population categories. Based on the private hunters’ handling of the slaughter waste these categories was further subdivided. In category A, slaughter waste was made unavailable to wildlife and in category B, the slaughter waste was left out in the forest (Table 3). All individuals sampled at hunting estates fell into Category 3A.

**Table 2:** The results from PCR analysis of tissue specimens originating from wild boars in 10 counties of Sweden

Tissue sampled	Total numbers analysed	Total numbers (%) positive for *Salmonella* spp.	Total numbers (%) positive for *Y. enterocolitica*	Total numbers (%) positive for *Y. pseudotuberculosis*
Tonsils	136	20 (14.7)	19 (14.0)	20 (14.7)
Submandibular lymph node	25	3 (12.0)	3 (12.0)	0 (0.0)
Other tissue from the throat region	14	6 (42.9)	4 (28.6)	0 (0.0)
Mesenteric lymph node	90	9 (10.0)	6 (6.7)	4 (4.4)
Faeces	90	7 (7.8)	4 (4.4)	2 (2.2)
Total	354	45 (12.7)	36 (10.2)	26 (7.3)

The animals were sampled at slaughter and the samples were analysed for the presence of *Salmonella* spp., *Y. enterocolitica* and *Y. pseudotuberculosis*

**Table 3:** Subdivision of the individual wild boars and the populations from where these wild boars where harvested, based on the answers in the questionnaire, into categories

Category	No of individuals within each category (%)	No of populations within each category (%)
1A*	2 (2.2)	1 (3.3)
1B^#^	14 (15.6)	7 (23.3)
2A*	7 (7.8)	3 (10.0)
2B^#^	14 (15.6)	9 (30.0)
3A*	48 (53.3)	7 (23.3)
3B^#^	5 (5.6)	3 (10.0)
Total	90 (100)	30 (100)

1 = Fulfils two or three of the following criteria, annual cull of < 15 wild boar/1,000 ha/year, < 3 feeders/1,000 ha or less than 5 years since establishment of the population. 2 = Fulfils two or three of the following criteria, annual cull of < 30 wild boar/1,000 ha/year, 3–5 feeders/1,000 ha or 5–7 years since establishment of the population. 3 = Fulfils two or three of the following criteria, annual cull of > 30 wild boar/1,000 ha/year, > 5 feeders/1,000 ha or more than 7 years since establishment of the population

* Slaughter-waste made unavailable to wildlife

^#^Slaughter-waste left out in the forest

A sample-size calculation for the obtained number of samples (n = 90) corrected for clustering within population, indicated that a difference of 23% between variables of interest would be detectable. The different population characteristics are shown in Table 1. In the questionnaire, all the different population characteristics were represented in the answers (Table 4).

**Table 4:** Samples from 90 wild boars, representing 30 local populations, and shot during the regular hunting seasons 2014–2016

Population characteristics	Total (%)	Negative	PCR-positive for *Salmonella* spp.	PCR-positive for *Y. enterocolitica*	*Y. pseudotuberculosis*
Feeding intensity (feeding places/10 km^2^)					
< 3	11 (12.2)	4 (36.4)	4 (36.4)	2 (18.2)	1 (9.1)
3–5	40 (44.4)	14 (35.0)	10 (25.0)	18 (45.0)	7 (17.5)
5–10	9 (10.0)	4 (44.4)	4 (44.4)	0 (0.0)	2 (22.2)
> 10	30 (33.3)	13 (43.3)	6 (20.0)	8 (26.7)	10 (33.3)
Years since establishment of population (years)					
< 3	1 (1.1)	0 (0.0)	0 (0.0)	1 (100.0)	0 (0.0)
3–5	5 (5.6)	0 (0.0)	2 (40.0)	4 (80.0)	2 (40.0)
5–7	10 (11.1)	4 (40.0)	5 (50.0)	3 (30.0)	0 (0.0)
7–10	5 (5.6)	0 (0.0)	3 (60.0)	2 (40.0)	2 (40.0)
> 10	69 (76.7)	32 (46.4)	14 (20.3)	18 (26.2)	16 (23.2)
Yearly harvest/10 km^2^ (animals)					
< 5	6 (6.7)	1 (16.7)	2 (33.3)	4 (66.7)	3 (50.0)
5–15	18 (20.0)	8 (44.4)	6 (33.3)	5 (27.8)	0 (0.0)
15–30	15 (16.7)	4 (26.7)	5 (33.3)	6 (40.0)	2 (13.3)
30–50	22 (24.4)	10 (45.5)	5 (22.7)	5 (22.7)	5 (22.7)
> 50	29 (32.2)	12 (41.4)	6 (20.7)	8 (27.6)	10 (34.5)
Handling of slaughter waste					
Made unavailable for wildlife	58 (64.4)	22 (37.9)	14 (24.1)	17 (29.3)	17 (29.3)
Left out in the forest	32^a^ (35.6)	13 (40.6)	10 (31.3)	11 (34.4)	3 (9.4)

The number of wild boars positive for *Salmonella* spp., *Y. enterocolitica* and *Y. pseudotuberculosis* as analysed by PCR, is specified in relation to each population characteristic as defined in a questionnaire

^a^Only samples submitted by hunters within this category (68% of hunter-submitted samples)

### PCR-analysis

In total, 107 (30.2%) of the 354 samples were PCR-positive, representing 55 (61.0%) individuals that were positive for at least one of the three enteropathogens examined. In the analysis of *Salmonella* spp., a mean Ct-value of 30.9 was obtained (range 17–39). In the analysis of *Y. enterocolitica,* a mean Ct-value of 30.5 was obtained (range 22–38), and in the analysis of *Y. pseudotuberculosis,* a mean Ct-value of 32.6 was obtained (range 22–39). Twenty-four individuals (26.7%) were positive for *Salmonella* spp., 28 (31.0%) were positive for *Y. enterocolitica* and 20 (22.0%) were positive for *Y. pseudotuberculosis* (Table 5). The number of individual samples and tissue that were positive for any of the three pathogens is shown in Table 2, and the distribution of the different population characteristics within each tentative risk factor is shown in Table 4.

**Table 5:** The results of the PCR-analysis of samples from 90 wild boars representing 30 populations given as % (numbers in brackets)

PCR-results	Individual wild boars positive	Local population with ≥ 1 positive wild boar
Positive for any pathogen	61.0% (55)	76.7% (23)
Positive for *Y. enterocolitica*	31.0% (28)	50.0% (15)
Positive for *Y. pseudotuberculosis*	24.4% (20)	46.7% (14)
Positive for *Salmonella* spp.	26.7% (23)	30.0% (9)

The samples were analysed for the presence of the pathogens *Y. enterocolitica*, *Y. pseudotuberculosis* and *Salmonella* spp. An animal was deemed positive when at least one specimen was positive in the PCR

### Statistical analysis

Spearman’s rank correlation test indicated that feeding intensity was strongly positively correlated with population density (ρ = 0.81, *P < 0.0001).

No significant risk factors were found for the presence of any of the investigated enteropathogens (*P *< 0.05). Results from the logistic regression models are presented in Table 6.

**Table 6:** Samples from 90 wild boars analysed by PCR for the presence of *Y. enterocolitica*, *Y. pseudotuberculosis* and *Salmonella* spp.

	OR for the presence of			
	Any enteropathogen (n* = 55)	*Y. enterocolitica* (n* = 28)	*Salmonella* spp. (n* = 24)	*Y. pseudotuberculosis* (n* = 20)
High population density (> 30 harvested/10 km^2^, n^#^ = 51)	0.80 (0.34–1.89)	0.33 (0.07–1.62)	0.61 (0.15–2.53)	*2.83* (0.93–8.65) *P *= 0.0672
High frequency of artificial feeding places (> 5 feeding places/10 km^2^, n^#^ = 39)	0.85 (0.36–2.01)	0.24 (0.04–1.33)	1.08 (0.23–4.98)	*2.39* (0.87–6.60) *P *= 0.0929
Well established population (> 10 years, n^#^ = 69)	*0.28* (0.07–1.11) *P* = 0.0693	*0.20* (0.03–1.19) *P* = 0.0769	*0.27* (0.07–1.05) *P *= 0.0591	1.28 (0.38–4.37)
Poor handling of slaughter waste (n^#^ = 32)	1.12 (0.46–2.71)	0.73 (0.24–2.23)	0.50 (0.10–2.46)	2.67 (0.81–8.81)

The odds ratio for individual’s being positive by PCR for each of the risk factors was calculated using the positive individuals as cases and the negative individuals as controls. Confidence intervals are given within brackets. The analysed risk factors were population density, frequency of artificial feeding places, age of population and handling of slaughter waste. All associations with P < 0.1 are presented in italic and with the associate *P*-value

* Number of positives

^#^Number of individuals within this risk factor category

## Discussion

The present study attempted to investigate presumptive risk factors associated with the presence of human enteropathogens in wild boar in Sweden. Such studies has previously not been reported. In this study, we focused on *Salmonella* spp. and *Y. enterocolitica* that are commonly isolated from humans with enteric disease [34], as well as *Y. pseudotuberculosis* that has been responsible for several recent outbreaks of disease presumably related to wildlife [35, 36].

The findings indicate that one or more of these enteropathogens are present in almost 80% of the Swedish populations investigated and in 60% of the individual wild boars sampled. The higher prevalence obtained in the present study, as compared to our previous results [3], could be a result of the wider geographical area represented in the samples, or due to a modified and refined analysis protocol [37]. The findings are also comparable to the prevalence demonstrated in other European countries [38, 39]. In line with previous reports [8], tonsils appeared to be the most suitable sample material for these analyses. An investigation of meat products of wild boar origin would be necessary to further investigate the implication of these results for the public health.

The present study identified no significant risk factors among those investigated. Thus, no recommended interventions can be made based on the findings in this study. The intended sampling of 150 animals would have been able to detect a difference of 18% in the variable of interest, whereas the obtained number of animals (n = 90) were able to detect a difference of 23%. Thus, this difference did not seem to have a major influence on the results. A slight tendency towards a higher risk for the presence of *Y. pseudotuberculosis* was seen for the risk factors “high population density “and “high frequency of artificial feeding”. However, since there was a high correlation between these variables confounding makes it difficult, with the current samples, to determine if any of these variables, on their own, could be a true risk factor. This is reflected by the similar magnitude in OR for the association between “high population density”, “high frequency of artificial feeding” and presence of *Y. pseudotuberculosis* (Table 6). Other factors such as closeness to infected farms or contaminated surface water was not recorded in the present study but may affect the presence of these enteropathogens [13, 40]. The tendency towards a lower presence of *Salmonella* spp. and *Y. enterocolitica* in older populations is surprising. Speculatively, this could be due to an acquired immunity within a resident population, since these pathogens are more likely to be found in younger animals [8]. To investigate this further, targeted sampling of various age categories of animals is needed.

The sampling relied on hunter’s willingness to submit samples, in order to obtain a wide geographical distribution and variation in population characteristics. However, only two-thirds of the planned number of samples were obtained. A possible explanation could be a reluctance among the hunters to perform the sampling due to a lack of knowledge on anatomy, although a revision of the sampling instructions were sent out during the course of the study to further improve the sample quality. Another reason could be an apprehension among hunters to contribute to a study possibly discrediting wild boars as a food resource, hence introducing a participating bias that might have influenced the results.

Samples from 47 animals were sent in from hunters originating from 25 different populations and 43 animals sampled came from an additional five populations on commercial hunting estates with access to a wild game handling establishment. In the statistical analysis, correction was made for the location of sampling, to preclude interference of unrecorded local factors.

All of the 51 animals from the high density population (yearly harvest of > 30 animals/10 km^2^/year) were from well-established populations (> 10 years since establishment; Table 4). In the present study, recently established populations and low population densities had no protective effect on the presence of the pathogens investigated. The use of artificial feeding places was common in most populations sampled and three out of the five commercial hunting estates had > 10 feeding places per 10 km^2^, while only two hunters reported such a high level of feeding. In Scandinavia, the availability of artificial feeding will probably cancel out the limiting effect of harsh winters and thus be the determining factor for population densities [41]. Crowding of wild boars at feeding places in the winter will occur, implying a possible opportunity for transmission of various infectious agents. The population density was also high at the hunting estates with three estates harvesting > 50 wild boars per 10 km^2^/year and the other two harvesting 30–50 wild boars per 10 km^2^/year, while only one of the hunters reported harvesting > 50 wild boar per 10 km^2^/year (Table 4 and data not shown).

The present study also showed that the slaughter waste is commonly left out in the forest, as two-thirds of the hunters adopted this routine. Clearly, this implies that there are areas in Sweden, where only minor parts of the slaughter waste (intestines) are left out in the forest (e.g. large hunting estates), while there are other areas where all slaughter waste (including head and tonsils) are available for wildlife. Although handling of slaughter waste was not identified as a risk factor in the present study, these remnants, available for scavengers such as red fox, corvid birds and rats [9, 42], could pose a risk for the spread of pathogens to the Swedish wild boar population and/or domestic animals. This risk is yet to be investigated.

Other factors, not included in the present study, may also be associated with the presence of enteropathogens in wild boar. For example, birds and rodents carrying pathogenic *Yersinia* spp. [9, 10] and *Salmonella* Typhimurium DT40 and DT56 [43] could hypothetically be associated with the presence of these pathogens in wild boar.

## Conclusions

With a fast growing and well-established population, the presence of human enteropathogens in wild boar will be difficult to manage by other means than good hygiene practices at slaughter and through biosecurity measures on the farms. Based on the present results, the effect of reducing population densities and number of artificial feeding places might be limited. However, these measures and the consequences from poor handling of slaughter-waste, as well as other presumptive risk factors, need to be further studied.

The high prevalences of enteropathogenic *Yersinia* spp. and *Salmonella* spp. found in the present study are a matter of concern for public health. However, no association was found with the population density, frequency of artificial feeding, the age of population, or the handling of slaughter waste. A correlation between feeding intensity and population density was seen and the practice of leaving slaughter-waste from wild boar out in the woods was found to be a common practise among hunters. Thus, the impact of these factors needs to be further studied.

## Abbreviations

BHI: brain heart infusion broth
BG: brilliant green agar
BPW: buffered peptone water
CIN: Cefsulodin–Irgasan–Novobiocin agar
Ct-value: cycle threshold value
OR: odds ratio
PCR: polymerase chain reaction
XLD: xylose lysine deoxycholate agar

## References

[1] Ruiz-Fons F (2017). A review of the current status of relevant zoonotic pathogens in wild swine (*Sus scrofa*) populations: changes modulating the risk of transmission to humans. Transbound Emerg Dis..

[2] Widén F (2010). Molecular epidemiology of hepatitis E virus in humans, pigs and wild boars in Sweden. Epidemiol Infect.

[3] Sanno A, Aspan A, Hestvik G, Jacobson M (2014). Presence of *Salmonella* spp., *Yersinia enterocolitica*, *Yersinia pseudotuberculosis* and *Escherichia coli* O157:H7 in wild boars. Epidemiol Infect.

[4] Wallander C, Frossling J, Vagsholm I, Uggla A, Lunden A (2015). *Toxoplasma gondii* seroprevalence in wild boars (*Sus scrofa*) in Sweden and evaluation of ELISA test performance. Epidemiol Infect.

[5] Pozio E (2004). *Trichinella pseudospiralis* foci in Sweden. Vet Parasitol.

[6] Lindblad M, Lindmark H, Thisted Lambertz S, Lindqvist R (2007). Microbiological baseline study of swine carcasses at Swedish slaughterhouses. J Food Protect..

[7] Laukkanen R, Martinez PO, Siekkinen K-M, Ranta J, Maijala R, Korkeala H (2008). Transmission of *Yersinia pseudotuberculosis* in the pork production chain from farm to slaughterhouse. Appl Environ Microbiol.

[8] Fredriksson-Ahomaa M, Wacheck S, Koenig M, Stolle A, Stephan R (2009). Prevalence of pathogenic *Yersinia enterocolitica* and *Yersinia pseudotuberculosis* in wild boars in Switzerland. Int J Food Microbiol.

[9] Backhans A, Fellström C, Lambertz ST (2011). Occurrence of pathogenic *Yersinia enterocolitica* and *Yersinia pseudotuberculosis* in small wild rodents. Epidemiol Infect.

[10] Niskanen T, Waldenström J, Fredriksson-Ahomaa M, Olsen B, Korkeala H (2003). virF-positive *Yersinia pseudotuberculosis* and *Yersinia enterocolitica* found in migratory birds in Sweden. Appl Environ Microbiol.

[11] Osterberg J, Ekwall SJ, Nilsson I, Stampe M, Engvall A, Wallgren P (2001). Eradication of *Salmonella* Yoruba in an integrated pig herd. Berl Munch tierarztl..

[12] Skov MN (2008). Transmission of *Salmonella* between wildlife and meat-production animals in Denmark. J Appl Microbiol.

[13] Mentaberre G, Porrero MC, Navarro-Gonzalez N, Serrano E, Domínguez L, Lavín S (2013). Cattle drive *Salmonella* infection in the wildlife–livestock interface. Zoonoses Public Hlth..

[14] Gortázar C, Ferroglio E, Höfle U, Frölich K, Vicente J (2007). Diseases shared between wildlife and livestock: a European perspective. Eur J Wildlife Res..

[15] Murray MH, Becker DJ, Hall RJ, Hernandez SM (2016). Wildlife health and supplemental feeding: a review and management recommendations. Biol Conserv.

[16] Brook RK, Wal EV, van Beest FM, McLachlan SM (2013). Evaluating use of cattle winter feeding areas by elk and white-tailed deer: implications for managing bovine tuberculosis transmission risk from the ground up. Prev Vet Med..

[17] Phillips B, Kelehear C, Pizzatto L, Brown G, Barton D, Shine R (2010). Parasites and pathogens lag behind their host during periods of host range advance. Ecology.

[18] Thurfjell H, Spong G, Ericsson G (2013). Effects of hunting on wild boar *Sus scrofa* behaviour. Wildlife Biol..

[19] Massei G (2015). Wild boar populations up, numbers of hunters down? A review of trends and implications for Europe. Pest Manag Sci.

[20] EC: Corrigendum to Regulation (EC) No 853/2004 of the European Parliament and of the Council of 29 April 2004 laying down specific hygiene rules for food of animal origin (OJ L 139, 30.4.2004). Official Journal of the European Union; 2004.

[21] EC: Regulation (EC) (2009). Regulation (EC) No 1069/2009 of the European Parliament and of the Council of 21 October 2009 laying down health rules as regards animal by-products and derived products not intended for human consumption and repealing Regulation (EC) No 1774/2002 (Animal by-products Regulation). Official Journal of the European Union..

[22] Swedish Board of Agriculture. Från skog till krog—Vilka hinder motverkar mer vildsvinskött på marknaden? (Swedish only). In: Karlsson AWCJ, editors. vol. 28: Swedish Board of Agriculture; 2013.

[23] Dohoo IR, Martin W, Stryhn HE (2003). Veterinary epidemiologic research.

[24] Truvé J, Lemel J, Söderberg B (2004). Dispersal in relation to population density in wild boar (*Sus scrofa*). Galemys.

[25] Schiemann DA (1979). Synthesis of a selective agar medium for *Yersinia enterocolitica*. Can J Microbiol.

[26] Anonymous (2002). ISO 6579:2002 Microbiology of food and animal feeding stuffs—Horizontal method for the detection of *Salmonella* spp.

[27] Lambertz ST, Nilsson C, Hallanvuo S (2008). TaqMan-based real-time PCR method for detection of *Yersinia pseudotuberculosis* in food. Appl Environ Microbiol.

[28] Lambertz ST, Nilsson C, Hallanvuo S, Lindblad M (2008). Real-time PCR method for detection of pathogenic *Yersinia enterocolitica* in food. Appl Environ Microbiol.

[29] Hoorfar J, Ahrens P, Rådström P (2000). Automated 5′ nuclease PCR assay for identification of *Salmonella enterica*. J Clin Microbiol.

[30] Engelmann M, Lagerkvist CJ, Gren IM (2016). Hunting value of wild boar in Sweden.

[31] Karlsson A. Magsäcksinnehåll och reproduktion hos vildsvin i Sverige. In: Matersthesis/Swedish University of agricultural sciences, Faculty of veterinary medicine and animal husbandry, Veterinary program, vol. 2014. Swedish University of Agricultural science; 2014. p. 64.

[32] Bates D, Mächler M, Bolker B, Walker S (2015). Fitting linear mixed-effects models using lme4. J Stat Softw..

[33] R Core Team (2018). R: A language and environment for statistical computing.

[34] European Food Safety A, European Centre for Disease P, Control (2016). The European Union summary report on trends and sources of zoonoses, zoonotic agents and food-borne outbreaks in 2015. EFSA Journal..

[35] Williamson DA (2016). Genomic insights into a sustained national outbreak of *Yersinia pseudotuberculosis*. Genome Biol Evol..

[36] Halkilahti J, Haukka K, Siitonen A (2013). Genotyping of outbreak-associated and sporadic *Yersinia pseudotuberculosis* strains by novel multilocus variable-number tandem repeat analysis (MLVA). J Microbiol Methods.

[37] Sannö A, Jacobson M, Sterner S, Thisted-Lambertz S, Aspán A (2018). The development of a screening protocol for *Salmonella* spp. and enteropathogenic *Yersinia* spp. in wildlife samples also generating MLVA—data for *Y. enterocolitica* and *Y. pseudotuberculosis*. J Microbiol Methods.

[38] Wacheck S, Fredriksson-Ahomaa M, König M, Stolle A, Stephan R (2010). Wild Boars as an important reservoir for foodborne pathogens. Foodborne Pathog Dis..

[39] Paulsen P, Smulders FJM, Hilbert F (2012). *Salmonella* in meat from hunted game: a central European perspective. Food Res Int.

[40] Cheyne BM, Van Dyke MI, Anderson WB, Huck PM (2010). The detection of *Yersinia enterocolitica* in surface water by quantitative PCR amplification of the ail and yadA genes. J Water Health..

[41] Oja R, Kaasik A, Valdmann H (2014). Winter severity or supplementary feeding—which matters more for wild boar?. Acta Theriol.

[42] Sørensen KK (2005). Acute toxoplasmosis in three wild arctic foxes (*Alopex lagopus*) from Svalbard; one with co-infections of *Salmonella Enteritidis* PT1 and *Yersinia pseudotuberculosis* serotype 2b. Res Vet Sci.

[43] Horton RA (2013). Wild birds carry similar *Salmonella enterica* serovar Typhimurium strains to those found in domestic animals and livestock. Res Vet Sci.

